# Case Report: A rare motor storm in lupus: opsoclonus-myoclonus-ataxia syndrome as an atypical presentation of severe systemic lupus erythematosus

**DOI:** 10.3389/fmed.2026.1816940

**Published:** 2026-07-30

**Authors:** Marco Vinicio Culqui-Sánchez, César Felipe Romero Carvajal, Daniela Rocío Di Capua Sacoto, Esteban Ortiz-Prado, Juan S. Izquierdo-Condoy

**Affiliations:** 1 Facultad de Salud y Bienestar, Pontificia Universidad Católica del Ecuador, Quito, Ecuador; 2 Servicio de Neurología, Hospital de Especialidades Eugenio Espejo, Quito, Ecuador; 3 One Health Research Group, Universidad de Las Americas, Quito, Ecuador

**Keywords:** autoimmune movement disorder, neuropsychiatric lupus, opsoclonus–myoclonus–ataxia syndrome, rituximab, systemic lupus erythematosus

## Abstract

**Background:**

Opsoclonus–myoclonus–ataxia syndrome (OMAS) is a rare hyperkinetic movement disorder characterized by opsoclonus, generalized myoclonus, and cerebellar ataxia. In adults, OMAS is most often reported in paraneoplastic or postinfectious settings, whereas autoimmune etiologies are less common.

**Case presentation:**

A 21-year-old woman with a 7-month history of SLE developed a 3-week subacute OMAS phenotype with opsoclonus, generalized myoclonus, and gait/limb ataxia, leading to loss of independent ambulation. CSF, microbiological testing, and brain MRI were unremarkable, and initial malignancy screening was negative. Severe lupus activity was documented (SLEDAI-2 K 27). She received methylprednisolone pulses, plasmapheresis, and rituximab, with progressive neurological recovery and a decrease in lupus activity to SLEDAI-2 K 4. At discharge, she ambulated independently with mild residual gait ataxia.

**Conclusion:**

This case expands the spectrum of neuropsychiatric SLE by illustrating OMAS as a predominantly motor phenotype. A structured evaluation to exclude structural, infectious, and malignant etiologies is essential, and early escalation of immunotherapy may be associated with substantial neurological recovery when OMAS occurs in the context of severe lupus activity.

## 1 Introduction

Systemic lupus erythematosus (SLE) is a chronic inflammatory autoimmune disease associated with genetic variants in HLA-DR2, HLA-DR3, IRF5, STAT4, PTPN22, ITGAM, TNFAIP3, and other genes. These variants confer immunological susceptibility and promote aberrant activation of B and T lymphocytes. SLE predominantly affects young women and manifests with a broad spectrum of clinical features (1–3). It can involve multiple organs and systems, and among its most complex manifestations is neuropsychiatric involvement, which is collectively termed neuropsychiatric lupus (NPSLE) (4). These manifestations include seizures, cognitive dysfunction, psychosis, peripheral neuropathies, and complex motor syndromes (5).

**Figure 1:**
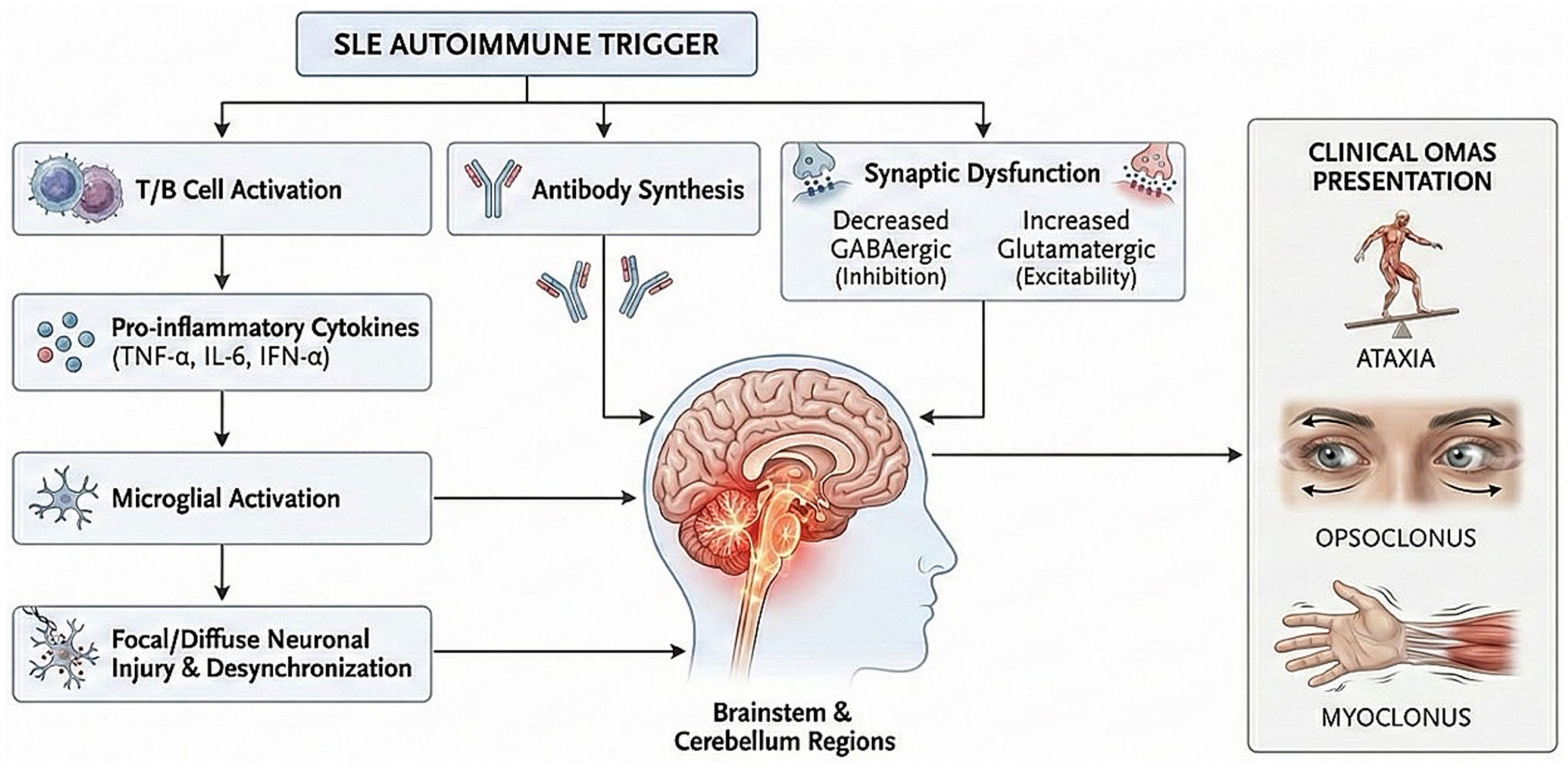
Opsoclonus–myoclonus–ataxia syndrome (OMAS) in systemic lupus erythematosus: etiologic framework and pragmatic diagnostic–therapeutic approach. The figure depicts the clinical trial of OMAS—opsoclonus, generalized myoclonus, and cerebellar ataxia—and situates it within a pragmatic workflow for patients with active SLE. It outlines the key etiologic categories to consider (paraneoplastic, postinfectious, and autoimmune), the recommended initial evaluation to exclude structural and infectious causes and screen for malignancy when clinically indicated, and the stepwise escalation of immunotherapy in suspected immune-mediated OMA.

Opsoclonus–myoclonus–ataxia syndrome (OMAS) is an uncommon hyperkinetic movement disorder characterized by the triad of rapid, multidirectional saccadic ocular oscillations (opsoclonus), generalized myoclonus, and cerebellar ataxia (6, 7). In adults, OMAS is most frequently associated with paraneoplastic (8, 9), or postinfectious etiologies (10, 11), whereas primary autoimmune causes are comparatively less common. Because OMAS likely reflects immune-mediated dysfunction of brainstem–cerebellar circuits, early differentiation among infectious, neoplastic, and autoimmune drivers is critical to guide timely therapy and optimize prognosis (12–14). The main etiologic categories and a pragmatic diagnostic–therapeutic framework are summarized in Figure 1.

The association between OMAS and systemic lupus erythematosus (SLE) has been reported only sporadically, suggesting that severe systemic autoimmunity may, in rare circumstances, be associated with this phenotype within the neuropsychiatric SLE (NPSLE) spectrum (12, 13, 15), Here, we describe a young woman with severe lupus activity who developed OMAS after exclusion of structural and infectious etiologies and without evidence of overt malignancy on initial evaluation, and who improved substantially after stepwise intensive immunotherapy. This case highlights a pragmatic diagnostic approach and supports early escalation of immunomodulatory therapy when OMAS occurs in the context of active SLE.

## 2 Case presentation

A 21-year-old Ecuadorian woman of Latin American mestizo ancestry with a 7-month history of systemic lupus erythematosus (SLE) initially presented with musculoskeletal and cutaneous manifestations. Previously, she had presented with cutaneous and articular manifestations and had been prescribed prednisone, hydroxychloroquine, mycophenolate mofetil (MMF), and methotrexate at a secondary-level healthcare institution prior to referral. The rationale for the combined use of MMF and methotrexate was not documented in the available medical records. In the months preceding admission, she reported irregular adherence and gastrointestinal intolerance to MMF, leading to temporary discontinuation.

Approximately 3 weeks before hospitalization, she developed progressively worsening holocranial headache with persistent nausea, occasional vomiting, photophobia, and gait instability. Over the ensuing days, symptoms progressed to include postural instability, impaired fine motor control, visuomotor incoordination, and involuntary multidirectional eye movements (“my eyes moving on their own”), followed by myoclonic jerks of the upper and lower extremities. The jerks increased in frequency and severity, ultimately precluding independent ambulation.

On admission to the neurology service, she was alert and oriented, hemodynamically stable, and without meningeal signs. Neurological examination showed multidirectional saccadic oscillations consistent with opsoclonus, predominantly distal generalized myoclonus, and marked limb and gait ataxia. Finger-to-nose and heel-to-shin testing demonstrated bilateral dysmetria. General physical examination revealed nonpruritic macular lesions on the trunk and extremities and tenderness and swelling involving the shoulders, elbows, knees, and ankles, consistent with active inflammatory arthritis.

Laboratory testing demonstrated leukopenia, mild anemia, and marked elevation of C-reactive protein. Antinuclear antibodies (ANA: 8.00) and anti–double-stranded DNA antibodies (dsDNA: 26.20) were positive. Complement levels were reduced (C3: 0.19; C4: 0.03), consistent with active lupus (Table 1).

**Table 1:** Laboratory findings at admission.

Parameter	Result	Reference range
White blood cells (/μL)	2.88	4,000–10,000
Lymphocytes (/μL)	650	1,000–4,000
Hemoglobin (g/dL)	11	12–16
C-reactive protein (mg/dL)	4.43	<0.5
Antinuclear antibodies (ANA index)	8.00	Negative <1.0
Anti–double-stranded DNA antibodies (IU/mL)	26.20	<15
Complement C3 (g/L)	0.19	0.90–1.80
Complement C4 (g/L)	0.03	0.10–0.40

Cerebrospinal fluid (CSF) analysis revealed clear and colorless fluid with 10 leukocytes/μL (96% mononuclear cells), no erythrocytes, glucose of 49.8 mg/dL, protein concentration of 36.9 mg/dL, and LDH of 14 U/L. Cytological examination was acellular and negative for malignancy. A repeat CSF evaluation showed 32 leukocytes/μL (94% mononuclear cells), glucose of 76 mg/dL, protein concentration of 27.7 mg/dL, and LDH of 12.9 U/L. Extensive microbiological investigation was negative, including Gram stain, India ink preparation, bacterial cultures, GeneXpert testing, and a multiplex meningitis/encephalitis PCR panel. No bacterial, viral, or fungal pathogens were detected, including *Haemophilus influenzae*, *Neisseria meningitidis*, *Listeria monocytogenes*, *Streptococcus pneumoniae*, *Escherichia coli* K1, cytomegalovirus, enterovirus, human herpesvirus 6, herpes simplex virus 1 and 2, varicella-zoster virus, parechovirus, or *Cryptococcus neoformans*/gattii.

Given the adult OMAS phenotype, a paraneoplastic evaluation was pursued. Tumor markers (CA-125, CA 19–9, carcinoembryonic antigen [CEA], and alpha-fetoprotein [AFP]) were negative. Paraneoplastic antibody testing by enzyme-linked immunosorbent assay (ELISA) was negative for anti-Yo and anti-Ri and positive for anti-Hu. Because anti-Hu (ANNA-1) is strongly associated with paraneoplastic neurologic syndromes and ELISA may yield false-positive results, confirmatory testing was requested; however, the result was not available during the acute hospitalization. Given the positive anti-Hu result, the patient was referred for longitudinal oncologic surveillance. At the time of manuscript preparation, no clinical, radiological, or laboratory evidence of malignancy had been identified.

Transvaginal ultrasonography showed an anteverted uterus of normal size and structure, with homogeneous myometrium and no adnexal masses; a small amount of fluid was noted in the rectouterine pouch. Contrast-enhanced whole-body computed tomography revealed no abnormalities.

Electroencephalography during spontaneous sleep (N1–N2) showed a normal background with vertex waves, K-complexes, and sleep spindles, without epileptiform activity. Brain magnetic resonance imaging revealed no structural abnormalities or findings suggestive of active pathology.

The SLEDAI-2 K score at admission was 27, indicating severe lupus activity. The baseline SLEDAI-2 K score reflected active neuropsychiatric involvement, inflammatory arthritis, cutaneous manifestations, serological activity (positive anti-dsDNA antibodies and hypocomplementemia), and hematological involvement. Given clinical features consistent with OMAS in the context of active SLE, intensive immunomodulatory therapy was initiated. She received five pulses of methylprednisolone followed by five sessions of plasmapheresis. Two intravenous doses of rituximab (1 g each, 14 days apart) were subsequently administered.

Neurological improvement was progressive. A modest response followed corticosteroids, with more marked motor recovery after the second rituximab infusion. The reduction in SLEDAI-2 K score reflected resolution of the neuropsychiatric, musculoskeletal, cutaneous, and hematological manifestations observed at presentation. The residual score of 4 was attributable to persistent serological activity, including positive anti-dsDNA antibodies and low complement levels.

**Figure 2:**
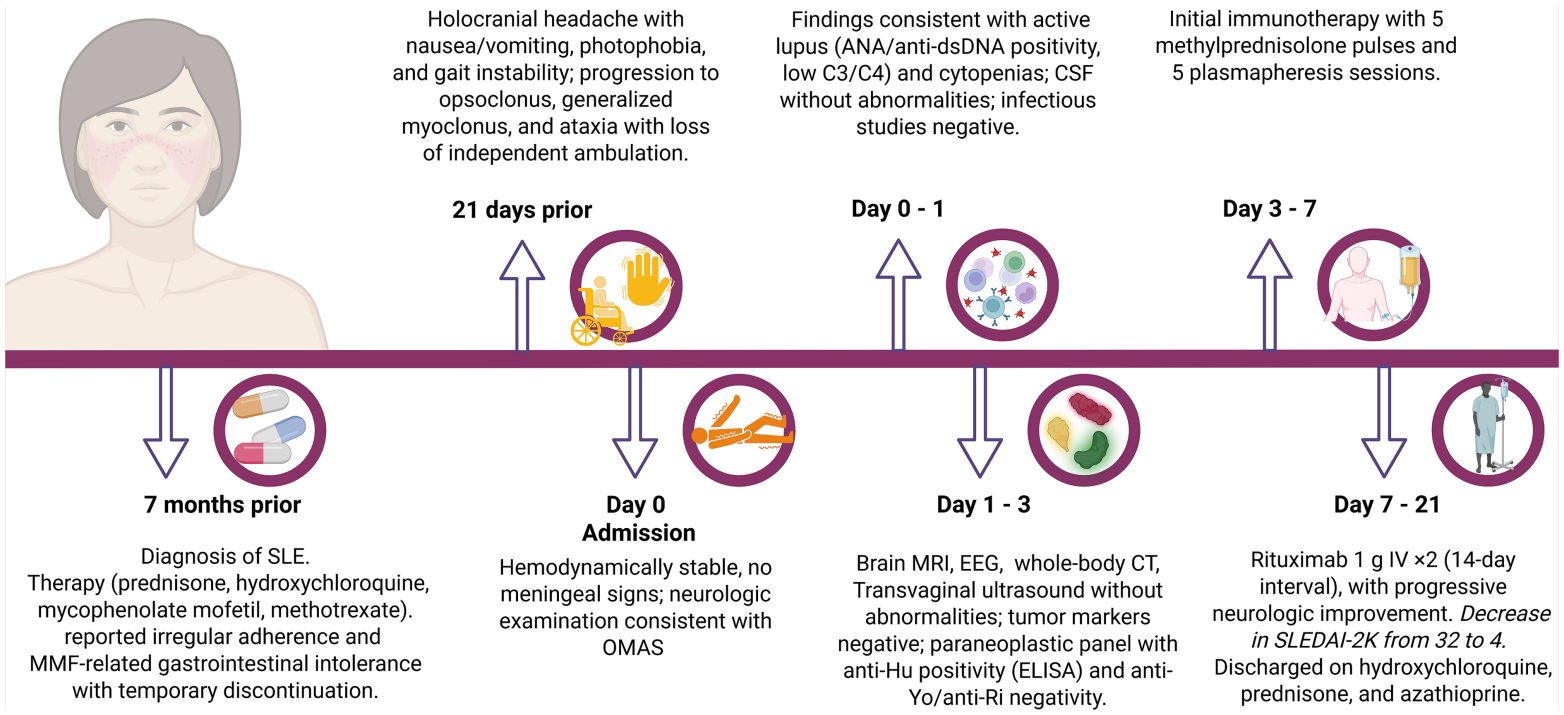
Timeline of the clinical course, diagnostic workup, and therapeutic interventions.

At discharge, she was able to ambulate independently and perform basic activities of daily living. She was discharged on hydroxychloroquine, prednisone, and azathioprine, with multidisciplinary outpatient follow-up including physical rehabilitation and neuropsychological evaluation (Figure 2).

### 2.1 Follow-up

The patient was followed for more than 12 months through three multidisciplinary outpatient evaluations by rheumatology and neurology services. During follow-up, she received a third dose of rituximab as part of maintenance immunomodulatory therapy. Progressive neurological recovery was observed, with complete resolution of ataxia and myoclonus and partial resolution of opsoclonus, enabling independent performance of activities of daily living. No new neuropsychiatric manifestations of SLE were documented, and lupus disease activity remained clinically controlled. Although a formal oncologic surveillance protocol was not implemented, no constitutional symptoms, laboratory abnormalities, imaging findings, or other clinical features suggestive of an underlying paraneoplastic process emerged during follow-up. Sustained clinical stability, including after the additional rituximab administration, was consistent with the benefit of ongoing immunomodulatory therapy; however, conclusions regarding the specific efficacy of rituximab cannot be drawn from a single observational case.

## 3 Discussion

This case illustrates OMAS occurring in the context of active SLE, an infrequent presentation with limited representation in the literature (16, 17). In adults, OMAS is more commonly reported in paraneoplastic or postinfectious settings; therefore, recognizing the possibility of an immune-mediated phenotype is clinically relevant, particularly when symptoms develop subacutely in a patient with known systemic autoimmunity. This report underscores the importance of maintaining a broad differential diagnosis and prioritizing a structured evaluation when prominent motor symptoms emerge in patients with SLE.

Headache was the earliest neurological symptom in this patient and preceded the development of opsoclonus, myoclonus, and ataxia by several weeks. Although headache is a common and nonspecific symptom in patients with systemic lupus erythematosus, its occurrence in the setting of marked serological activity and subsequent neurological manifestations raises the possibility that it represented an early neuropsychiatric manifestation of active disease. Nevertheless, a direct causal relationship cannot be established from an individual case.

Onconeural antibodies are not typically associated with SLE; however, isolated reports describe their detection in patients with motor neurological manifestations without an identifiable neoplasm (18). In this context, anti-Hu positivity—especially when detected by ELISA—should be interpreted cautiously given the possibility of false-positive results and the strong association of anti-Hu (ANNA-1) with paraneoplastic neurological syndromes. Although OMAS has been described more frequently in neonatal lupus and pediatric autoimmune disorders, reports in adult patients with systemic lupus erythematosus remain exceedingly rare. Consequently, the available evidence is limited to isolated case reports and small case series, making diagnostic and therapeutic decisions particularly challenging. The present case contributes additional evidence to the growing recognition that OMAS may occur in association with active SLE in adults.

Methotrexate-related neurotoxicity was also considered in the differential diagnosis. Although neurological adverse events have been reported with methotrexate, they occur predominantly in the setting of high-dose or intrathecal administration and are uncommon at conventional rheumatologic doses. Furthermore, the patient had been receiving methotrexate before symptom onset without documented neurological complications, and brain MRI did not reveal findings typically associated with methotrexate-induced neurotoxicity. Consequently, methotrexate was considered a less likely explanation for the observed neurological syndrome.

A positron emission tomography (PET) study was not performed and may have provided additional sensitivity for detecting an occult neoplasm. This represents a limitation of the present report. Nevertheless, extensive baseline evaluation, including contrast-enhanced whole-body computed tomography, transvaginal ultrasonography, tumor marker assessment, cerebrospinal fluid cytology, and prolonged clinical follow-up exceeding 12 months, failed to identify evidence of an underlying malignancy.

The patient’s neurological recovery occurred during a stepwise escalation of immunotherapy that included corticosteroids, plasmapheresis, and rituximab. Because these interventions were administered sequentially, the relative contribution of each treatment cannot be determined. Therefore, the observed clinical improvement should be interpreted as associated with the overall immunomodulatory strategy rather than with any single therapeutic agent.

Also noteworthy was the systematic exclusion of infectious, structural, and neoplastic causes through detailed cerebrospinal fluid analysis, cytological examination, microbiological studies, neuroimaging, EEG, and extensive laboratory testing, which strengthened the diagnostic consideration of an immune-mediated OMAS occurring in the context of active SLE.

The stepwise escalation of immunomodulatory therapy was associated with marked neurological recovery and no severe adverse events. A key limitation is that confirmatory testing for the onconeural antibody result was not immediately available during acute decision-making, and long-term follow-up remains essential to monitor for relapse and to reassess for occult malignancy.

Only a small number of reports have linked OMAS with SLE (18, 19), and responses to immunosuppressive therapy appear variable (9, 20). Compared with more common neuropsychiatric SLE presentations—where seizures and cognitive or psychiatric symptoms predominate (21–24), this case highlights a predominantly motor phenotype that can pose diagnostic challenges. These features support a comprehensive diagnostic approach integrating neuroimaging and cerebrospinal fluid studies to exclude structural and infectious causes, malignancy screening when clinically indicated, and consideration of immune-directed therapy when active lupus is present.

Regarding treatment, clinical responses to corticosteroids alone are heterogeneous in published cases, and B-cell–depleting therapy may be considered in severe or refractory motor syndromes (15). In our patient, improvement after escalation from high-dose corticosteroids and plasmapheresis to rituximab supports a stepwise strategy in selected cases, although optimal sequencing cannot be inferred from a single observation (15, 25, 26).

## 4 Conclusion

OMAS is a rare neurological disorder that may occur in association with active systemic lupus erythematosus (SLE). In the present case, an extensive diagnostic evaluation excluded structural and infectious causes, while the initial malignancy assessment was negative. Although the coexistence of severe lupus activity and OMAS suggests a possible immune-mediated association, a definitive causal relationship cannot be established from a single case report.

The patient’s neurological improvement occurred during stepwise intensive immunotherapy with corticosteroids, plasmapheresis, and rituximab, and was accompanied by a marked reduction in lupus disease activity. These findings support consideration of immune-directed treatment strategies in selected patients presenting with OMAS in the context of active SLE.

This case expands the recognized spectrum of neuropsychiatric SLE by highlighting a predominantly motor phenotype and underscores the importance of a comprehensive diagnostic approach, including exclusion of infectious, structural, and malignant etiologies. Continued clinical follow-up remains important to monitor for relapse and to reassess for occult malignancy when clinically indicated.

## Data Availability

The original contributions presented in the study are included in the article/supplementary material, further inquiries can be directed to the corresponding author.
